# Chlamydial histone homologs control developmental fitness in the next infection cycle

**DOI:** 10.1128/msphere.00358-26

**Published:** 2026-06-11

**Authors:** Yuxuan Wang, Matthew Pan, Temitope V. Coker, Jing Wang, Lingling Wang, Yaeun Jung, Guangming Zhong, Huizhou Fan

**Affiliations:** 1 Department of Pharmacology, Robert Wood Johnson Medical School, Rutgers University 214911, Piscataway, New Jersey, USA; 2 Jiangsu Agri-animal Husbandry Vocational College 118356, Taizhou, China; 3 Department of Microbiology and Immunology, University of Texas Health San Antonio 557944, San Antonio, Texas, USA; University of Minnesota Twin Cities, Minneapolis, Minnesota, USA

**Keywords:** *Chlamydia*, *Chlamydia trachomatis*, bacterial histone, *hctA*, *hctB*, hc1, hc2

## Abstract

The unique chlamydial developmental cycle comprises three stages: primary differentiation of infectious elementary bodies (EBs) into reticulate bodies (RBs), RB replication, and secondary differentiation into progeny EBs. Extensive chromosome remodeling during RB-to-EB differentiation is thought to be mediated by the histone homologs HctA and HctB. Here, we used an inducible CRISPR interference system to knock down *hctA*, *hctB*, or both genes in *Chlamydia trachomatis*. Surprisingly, knockdown of either histone gene alone or in combination caused only modest reductions in EB yield and did not prevent nucleoid condensation during the parental developmental cycle. In contrast, progeny EBs generated under histone knockdown conditions were markedly impaired in their ability to initiate subsequent infections. Secondary cultures initiated with HctA-deficient EBs exhibited a delay in primary EB-to-RB differentiation, whereas combined repression of *hctA* and *hctB* caused both delayed differentiation and persistently reduced genome accumulation, indicative of impaired RB formation and subsequent growth. Repression of *hctB* alone did not measurably affect genome replication in secondary cultures. Together, these findings reveal that chromosome organization established during EB maturation governs developmental fitness in the next infection cycle, uncovering a transgenerational role for chlamydial histone homologs.

**IMPORTANCE:**

Chlamydial histone homologs HctA and HctB are unusual among bacterial DNA-binding proteins in that they share sequence homology with mammalian histones and are developmentally regulated during the formation of infectious particles. Here, we show that reduced expression of HctA and HctB has only modest effects on visible nucleoid condensation and EB production, consistent with partial functional redundancy between the two histones and suggesting that additional nucleoid factors contribute to EB chromosome compaction. In contrast, deficiency of HctA and HctB during EB maturation has profound consequences in the next infection cycle, impairing completion of primary EB-to-RB differentiation and subsequent RB growth. These findings reveal a previously unrecognized transgenerational role for chlamydial histone homologs and establish chromosome organization during EB maturation as a key determinant of developmental fitness across infection cycles.

## INTRODUCTION

*Chlamydia* is an obligate intracellular bacterium whose pathogenic success depends on a biphasic developmental cycle that alternates between two morphologically and physiologically distinct forms: the infectious elementary body (EB) and the replicative reticulate body (RB) (1–3). EBs are small, electron-dense particles with highly compacted nucleoids, adapted for extracellular survival and host-cell entry, whereas RBs are larger and fragile, with less dense nucleoids consistent with active transcription and DNA replication (1–3). Following internalization, EBs differentiate into RBs within a membrane-bound inclusion, undergo multiple rounds of division, and subsequently redifferentiate into progeny EBs that are released to infect new host cells (1–6).

The dramatic nucleoid remodeling that accompanies RB-to-EB differentiation is thought to be mediated by two developmentally regulated histone homologs, HctA (Hc1) and HctB (Hc2) (7–11), hereafter referred to as histones for simplicity. Both proteins bind DNA and repress transcription *in vitro* (12–14), and their abundance increases late in the developmental cycle as EBs form (15, 16). However, the two genes are regulated differently. *hctA* transcripts accumulate earlier in infection but are translationally silenced by the small RNA *ihtA* until late stages, whereas *hctB* is expressed primarily during terminal RB-to-EB differentiation (17, 18).

Early heterologous-expression studies revealed strikingly distinct phenotypes for the two proteins. Overproduction of HctA in *Escherichia coli* causes rapid nucleoid condensation and severe growth inhibition, whereas HctB overexpression induces the formation of unusual coil-like DNA structures whose relevance to chlamydial chromosome organization remains unclear (7, 8, 10). These divergent phenotypes, together with the distinct developmental regulation of the two genes, suggested that HctA and HctB might perform non-identical biological functions in *Chlamydia* itself, functions that could not be resolved until genetic tools became available to perturb histone expression directly in the native pathogen.

Beyond their proposed role in genome condensation, it is unclear whether histone-mediated nucleoid organization contributes more broadly to developmental progression. In particular, it remains unknown whether chromosome states established during EB maturation serve solely structural roles in genome compaction or also influence subsequent developmental transitions. Given the obligate differentiation cycle of *Chlamydia*, nucleoid architecture established in EBs could, in principle, determine the efficiency with which these particles reinitiate growth following infection. To address these questions, we used inducible CRISPR interference to selectively reduce expression of *hctA*, *hctB*, or both genes during chlamydial development and examined their roles in EB formation and subsequent infection cycles. Our findings reveal that histone function extends beyond structural roles in genome condensation to influence developmental progression across infection cycles.

## RESULTS

### CRISPRi enables efficient and specific knockdown of *hctA* or *hctB*

**Fig 1:**
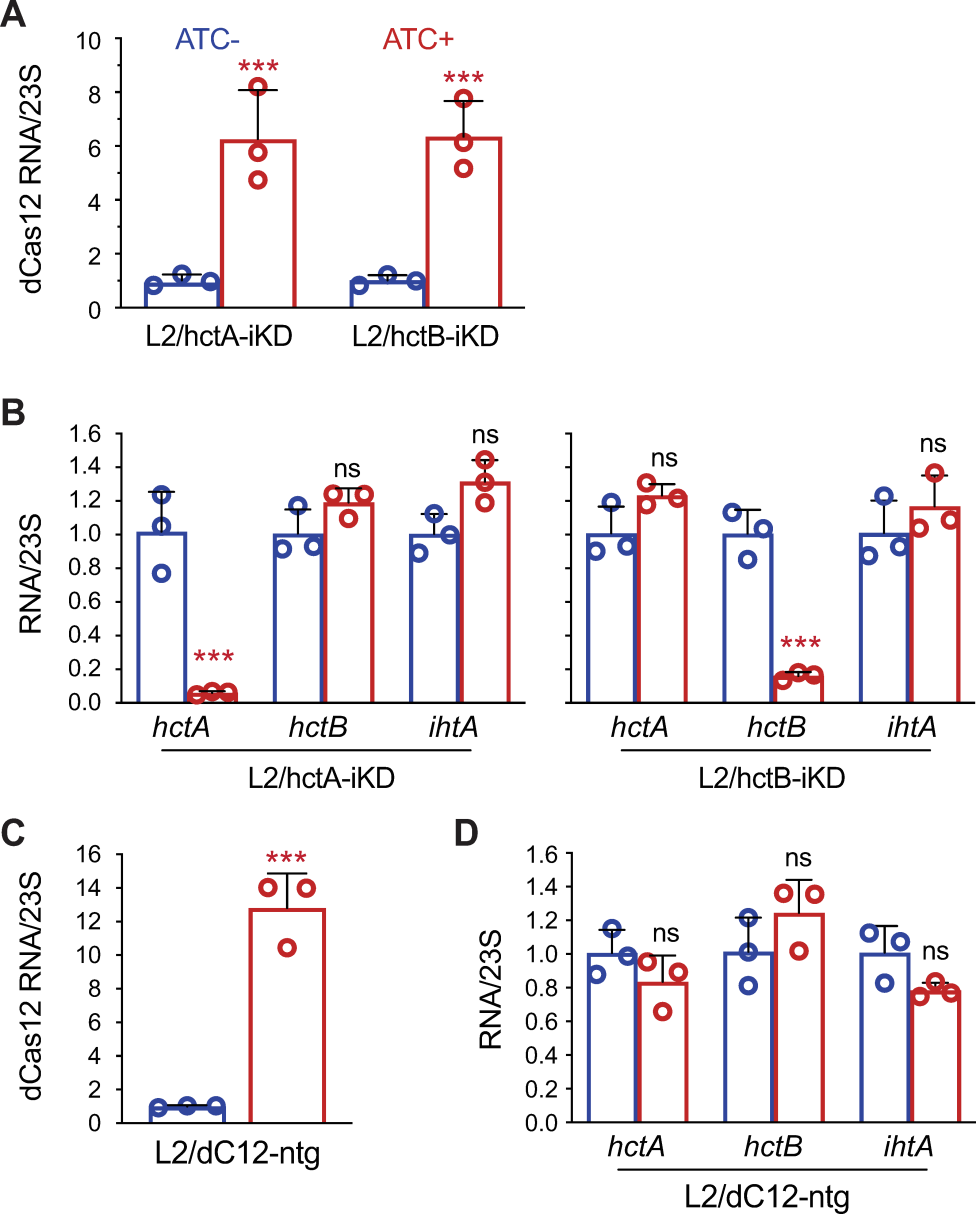
ATC induces ddCas12 expression and selectively reduces histone transcripts in L2/hctA-iKD and L2/hctB-iKD. (**A**) ATC-induced ddCas12 mRNA expression in L2/hctA-iKD and L2/hctB-iKD. (**B**) ATC-dependent reduction of the targeted histone transcript in L2/hctA-iKD and L2/hctB-iKD, with no effect on the non-targeted histone or *ihtA*. (**C**) ATC-induced ddCas12 mRNA expression in the control strain L2/ddCas12-ntg. (**D**) Unchanged RNA levels of *hctA, hctB,* and *ihtA* following ATC treatment in L2/ddCas12-ntg. (**A– D**) ATC was added at 0 hpi, total RNA was isolated at 24 hpi, and transcript abundance was determined by RT-qPCR and normalized to 23S rRNA. Data represent three independent biological replicates, with individual points shown. Statistical significance relative to ATC-free cultures is indicated (***, *P* < 0.001; ns, not significant).

To examine the roles of chlamydial histones during late development, we generated *C. trachomatis* L2 strains L2/hctA-iKD and L2/hctB-iKD, which express ATC-inducible ddCas12 and guide RNAs targeting either *hctA* or *hctB*. To determine knockdown efficiency, anhydrotetracycline (ATC) was added at 0 hour postinoculation (hpi), and transcript levels were quantified at 26 hpi, corresponding to the early late developmental phase. Under these conditions, ATC induction increased ddCas12 transcript abundance by approximately sixfold (Fig. 1A), resulting in strong knockdown of the targeted histone genes: *hctA* and *hctB* transcripts were reduced by 94% and 84% in L2/hctA-iKD and L2/hctB-iKD cultures, respectively, relative to ATC-free controls (Fig. 1B). Silencing of one histone gene did not affect the expression of the other and did not alter levels of *ihtA*, a small RNA that binds *hctA* mRNA to inhibit its translation (Fig. 1B). As expected, in the non-targeting control L2/ddCas12-ntg strain, ATC induced ddCas12 expression (Fig. 1C) without affecting the transcript levels of *hctA, hctB,* or *ihtA* (Fig. 1D). Collectively, these results demonstrate that CRISPRi enables selective and robust knockdown of individual histone genes during late development.

### Single-gene histone knockdowns do not impair RB replication but modestly reduce EB production

**Fig 2:**
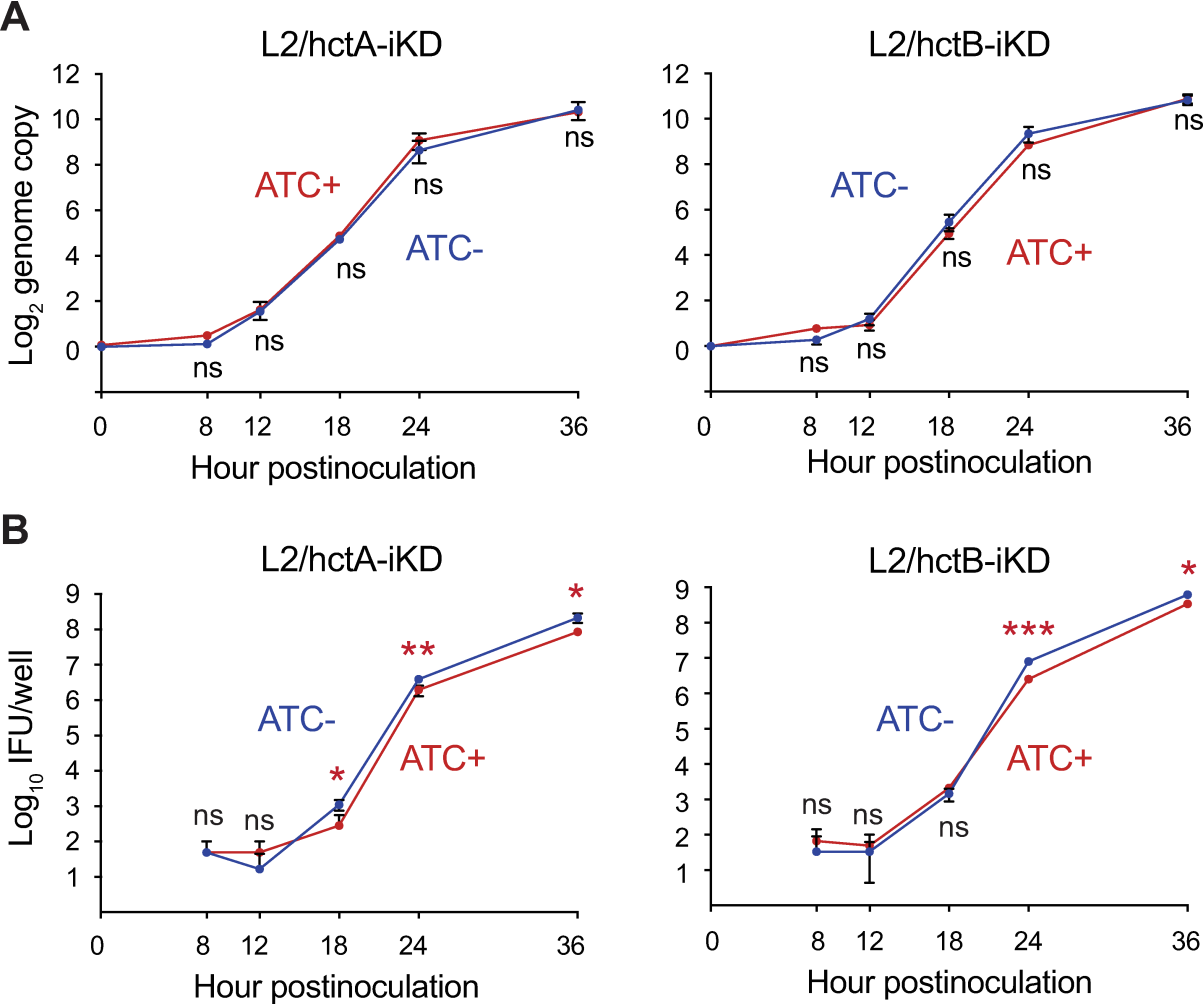
Single-gene knockdown of *hctA* or *hctB* does not measurably affect genome replication but modestly reduces EB production. (**A**) Genome copy number was determined by qPCR at the indicated time points in L2/hctA-iKD and L2/hctB-iKD cultures grown with or without ATC. Differences between ATC-treated and ATC-free cultures were not statistically significant at any time point for either strain. (**B**) Infectious progeny were measured by IFU assays at the indicated time points in L2/hctA-iKD and L2/hctB-iKD cultures grown with or without ATC. Data represent three independent biological replicates. Statistical significance for differences between ATC-treated and ATC-free cultures is indicated (*, *P* < 0.05; **, *P* < 0.01; *** *P* < 0.001; ns, not significant).

Because HctA and HctB are developmentally regulated and implicated primarily in late-stage genome condensation (7, 8), we predicted that their knockdown would not strongly affect RB replication but would compromise EB production. Indeed, in cultures grown with ATC, the genome replication kinetics of both L2/hctA-iKD and L2/hctB-iKD were indistinguishable from those of ATC-free controls (Fig. 2A). Surprisingly, IFU assays revealed that ATC-induced knockdown in L2/hctA-iKD and L2/hctB-iKD had modestly reduced EB yields, typically by only two- to threefold (Fig. 2B). These effects were far weaker than those reported for the inhibition of essential late developmental regulators (19, 20), suggesting that *Chlamydia* tolerates large reductions in histone expression during EB biogenesis.

### Histone gene knockdowns do not prevent EB morphogenesis or nucleoid condensation

**Fig 3:**
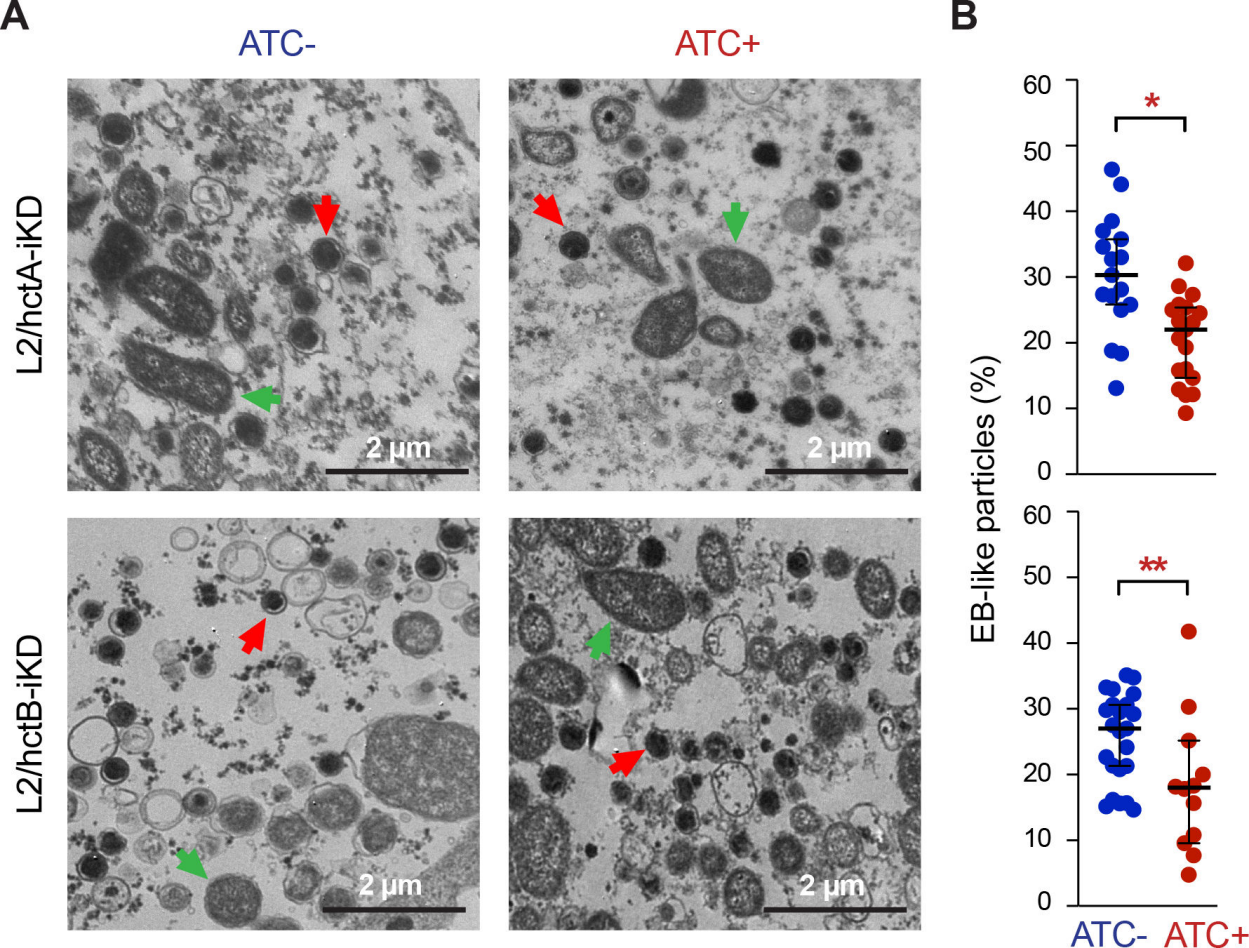
EB nucleoid condensation is preserved after ATC-induced *hctA* or *hctB* knockdown. (**A**) Representative transmission electron micrographs of L2/hctA-iKD and L2/hctB-iKD cultures grown with ATC added at 0 hpi or without ATC. Samples were collected at 36 hpi. Representative RB-like and EB-like particles are indicated by green and red arrows, respectively. (**B**) Quantification of EB-like particles as a percentage of total chlamydial particles in TEM fields. Data represent individual TEM fields, with bars indicating the mean ± SD. Statistical significance between ATC-treated and ATC-free cultures is indicated (*, *P* < 0.05; **, *P* < 0.01).

Because IFU assays indicated that histone knockdown still permitted the production of infectious progeny, we asked whether these particles corresponded to morphologically authentic EBs with condensed genomes. ATC-free and ATC-treated cultures of L2/hctA-iKD and L2/hctB-iKD were, therefore, examined by transmission electron microscopy (TEM) at 36 hpi. Similar to ATC-free cultures, ATC-treated cultures contained both RBs, characterized by larger size and relatively low electron density, and EB-like particles with smaller size and high electron density (Fig. 3A). EB-like particles with electron-dense nucleoids were readily detected under both knockdown conditions (Fig. 3A). Quantification showed that ATC treatment reduced the proportion of EB-like particles among total chlamydial particles in both knockdown strains (Fig. 3B), consistent with the modest reductions in infectious EB yield measured by IFU assays. These findings indicate that knockdown of either histone gene alone reduces the efficiency of EB morphogenesis but does not abolish gross EB formation or visible nucleoid condensation during late developmental stages.

### Knockdown of both *hctA* and *hctB* still has a limited effect on EB formation

**Fig 4:**
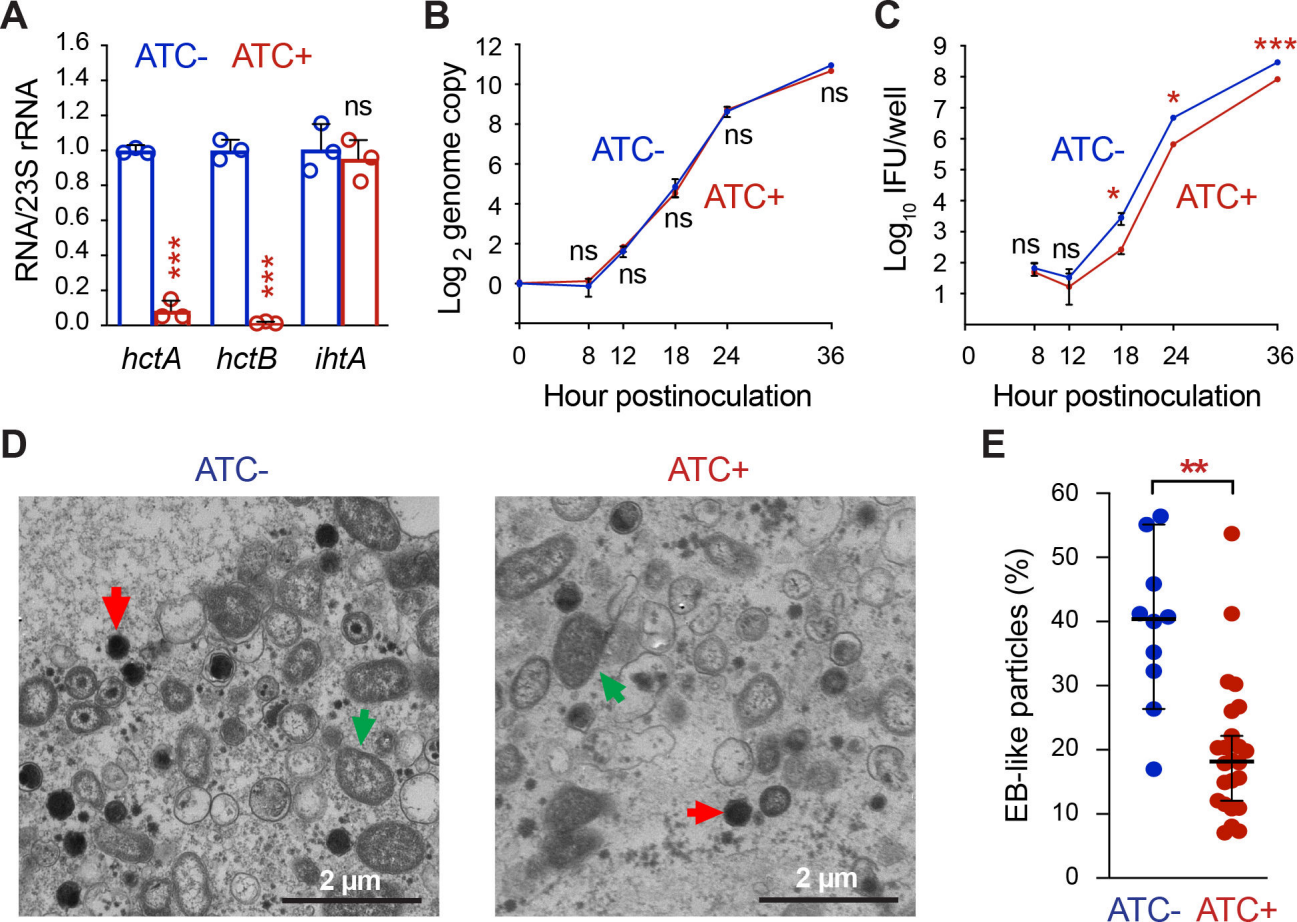
Knockdown of both *hctA* and *hctB* modestly reduces EB production while preserving nucleoid condensation. (**A**) ATC-dependent reduction of *hctA* and *hctB* transcripts but not *ihtA* levels in L2/hctAB-iKD. Data represent three independent biological replicates, with individual points shown. (**B**) Genome copy-number changes over time in L2/hctAB-iKD cultures grown with or without ATC. (**C**) EB production measured by IFU assays in L2/hctAB-iKD cultures with or without ATC. (**B and C**) Data represent three independent biological replicates. (**A–C**) RNA for RT-qPCR was isolated at 24 hpi. Statistical significance between ATC-treated and ATC-free cultures is indicated in the graphs. (**D**) Representative transmission electron micrographs of L2/hctAB-iKD-infected cells cultured with or without ATC. Samples were collected at 36 hpi. Representative RB-like and EB-like particles are indicated by green and red arrows, respectively. (**E**) Quantification of EB-like particles as a percentage of total chlamydial particles in TEM fields. Data represent individual TEM fields, with bars indicating the mean ± SD. Statistical significance between ATC-treated and ATC-free cultures is indicated (*, *P* < 0.05; **, *P* < 0.01; ***, *P* < 0.001; ns, not significant). (**A–E**) ATC was added at 0 hpi.

The limited phenotypes observed after single-gene knockdown (Fig. 2 and 3) suggested that HctA and HctB might perform overlapping functions during EB formation. To test redundancy, we generated a dual-targeting strain (L2/hctAB-iKD) expressing guide RNAs targeting both histone genes. ATC treatment reduced *hctA* and *hctB* transcript levels by 88% and 97%, respectively (Fig. 4A).

Similar to the single-gene knockdowns in L2/hctA-iKD and L2/hctB-iKD, genome replication kinetics in L2/hctAB-iKD remained indistinguishable between ATC-free and ATC-treated cultures (Fig. 4B). EB yields in ATC-treated L2/hctAB-iKD cultures were reduced slightly more than in ATC-treated L2/hctA-iKD and L2/hctB-iKD; however, the decreases remained less than 10-fold at each time point examined (Fig. 4C), supporting the conclusion that the effect of combined histone gene knockdown on EB formation was still modest. TEM further showed that EB-like particles with electron-dense nucleoids remained readily detectable in L2/hctAB-iKD cultures under dual-knockdown conditions (Fig. 4D). Quantification of TEM fields showed that ATC treatment reduced the proportion of EB-like particles among total chlamydial particles in L2/hctAB-iKD cultures (Fig. 4E), consistent with the reduction in infectious EB yield measured by IFU assays. These findings indicate that simultaneous knockdown of both histone genes does not substantially exacerbate the phenotypes observed after single-gene knockdown and that RB replication and EB morphogenesis remain largely intact despite severe reductions in histone transcript levels.

### Histone deficiency during EB formation compromises development in the next cycle

Although histone knockdown caused only limited defects during the initial developmental cycle, we noticed that inclusions were consistently smaller during IFU assays when secondary cultures were inoculated with EBs harvested from ATC+ L2/hctA-iKD and ATC+ L2/hctAB-iKD primary infections even though all secondary cultures were grown without ATC. This observation prompted us to examine systematically whether histone deficiency during EB formation impaired the ability of progeny EBs from all three knockdown strains to initiate subsequent infections by using the plasmid-encoded mKate reporter to quantify inclusion area and bacterial fluorescence intensity and by measuring genome replication kinetics in secondary cultures.

**Fig 5:**
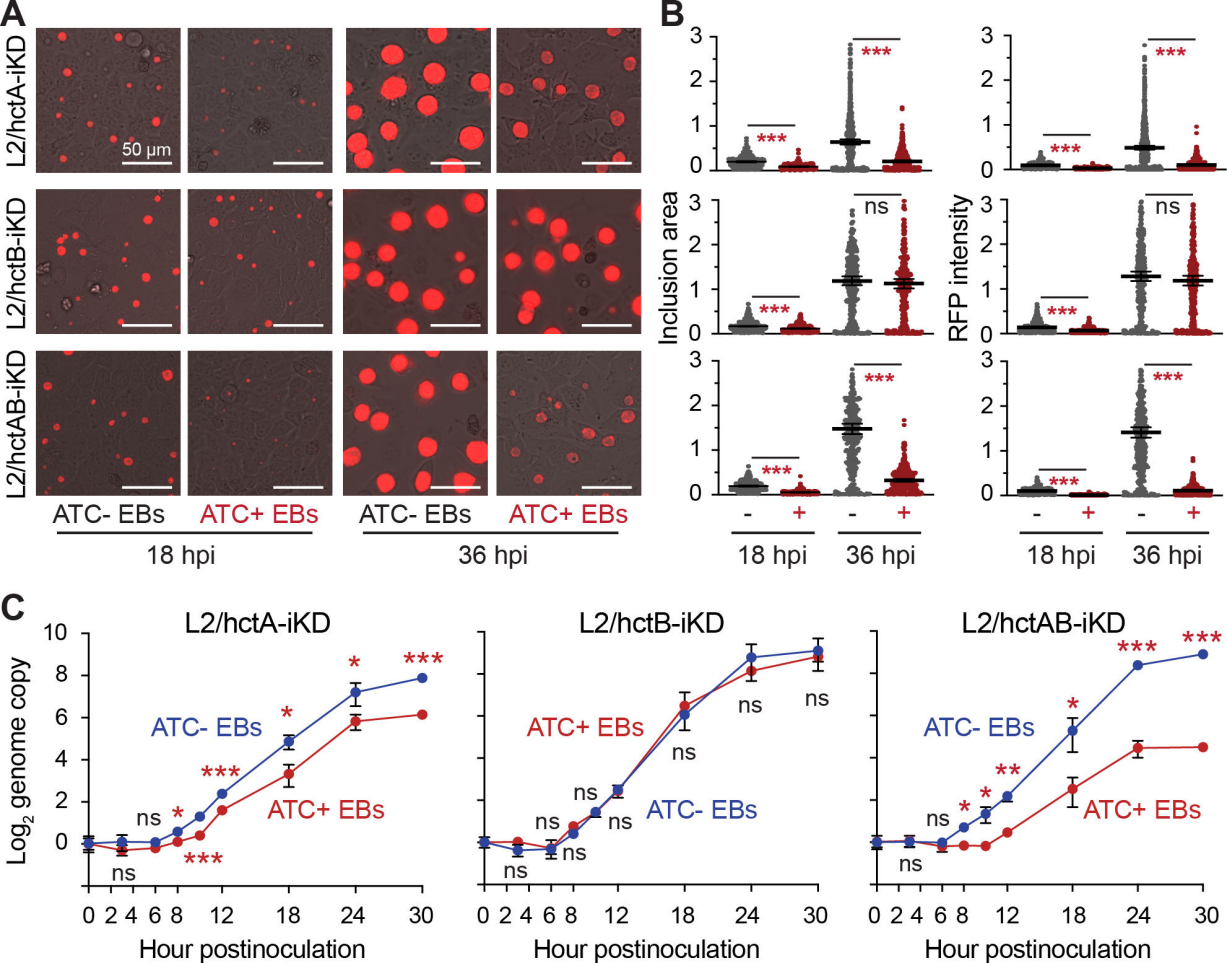
Histone deficiency during EB formation impairs development in secondary infections. (**A**) Representative fluorescence micrographs of secondary cultures infected with ATC− EBs or ATC+ EBs (EBs harvested from primary cultures grown without or with ATC, respectively) of L2/hctA-iKD, L2/hctB-iKD, and L2/hctAB-iKD at 18 and 36 hpi. (**B**) Quantification of inclusion area and mKate fluorescence intensity in secondary cultures at the indicated time points. “−” and “+” denote ATC− EBs or ATC+ EBs, respectively. (**C**) Genome copy number over time in secondary cultures infected with ATC− EBs or ATC+ EBs of L2/hctA-iKD, L2/hctB-iKD, and L2/hctAB-iKD. Data represent three independent biological replicates. (**B and C**) Statistical significance between cultures is indicated (*, *P* < 0.05; **, *P* < 0.01; ***, *P* < 0.001; ns, not significant). .

Representative fluorescence images at 18 and 36 h postinoculation showed that inclusions formed by ATC+ L2/hctA-iKD EBs were even smaller and dimmer than those formed by ATC− L2/hctA-iKD EBs, whereas inclusions formed by ATC+ L2/hctB-iKD EBs appeared largely similar to the corresponding ATC− controls (Fig. 5A). Quantification confirmed that the reductions in inclusion area and mKate intensity were more severe for cultures inoculated with ATC+ L2/hctAB-iKD EBs than for those inoculated with ATC+ L2/hctA-iKD EBs, while ATC+ L2/hctB-iKD EBs produced only a modest, transient decrease in inclusion area and mKate intensity at 18 h (Fig. 5B).

Quantification of genome copy numbers further resolved these phenotypes mechanistically (Fig. 5C). In cultures inoculated with ATC− EBs (EBs harvested from primary cultures grown without ATC), genome copy numbers began to increase by 8 h postinoculation, reflecting the completion of the primary EB-to-RB differentiation and the onset of detectable RB replication. In contrast, cultures inoculated with ATC+ L2/hctA-iKD EBs (EBs harvested from primary cultures grown with ATC) exhibited little increase through 12 h postinoculation, indicating a delay in the completion of primary EB-to-RB differentiation and the onset of detectable RB replication. After this initial lag, genome accumulation in cultures seeded with ATC+ L2/hctA-iKD EBs proceeded at a reduced rate compared with ATC− EB controls, producing a progressively widening divergence between the genome copy-number trajectories of secondary cultures initiated with ATC+ EBs and those initiated with ATC− EBs (Fig. 5C, left). By comparison, genome copy kinetics in cultures inoculated with ATC+ L2/hctB-iKD EBs closely overlapped those of ATC− EB controls throughout the time course (Fig. 5C, middle). Strikingly, cultures inoculated with ATC+ L2/hctAB-iKD EBs displayed the most severe defects: genome copy numbers remained near baseline until at least 12 h postinoculation and subsequently increased at a substantially reduced rate, resulting in persistently lower genome copy numbers at later time points (Fig. 5C, right). Together, these data indicate that histone deficiency during EB formation imposes a strong transgenerational defect in the establishment of a developmentally competent state required for efficient EB-to-RB differentiation and subsequent RB growth, with HctA governing the timing of RB emergence and HctB becoming functionally critical for robust replication when HctA is depleted.

## DISCUSSION

In this study, we used inducible CRISPRi to define the roles of the chlamydial histones HctA and HctB during late differentiation and across infection cycles. Although substantial reduction of histone transcripts had only modest effects on EB formation and did not prevent nucleoid condensation, histone deficiency during EB maturation markedly impaired developmental progression in the subsequent infection cycle. These findings indicate that nucleoid organization established during EB formation is not merely structural but establishes a chromosome state that governs the efficiency of EB-to-RB reactivation and RB growth in the next cycle, thereby determining developmental fitness across infection cycles.

The apparent dissociation between preserved nucleoid condensation and impaired developmental fitness indicates that bulk genome compaction and higher-order nucleoid organization are functionally distinct in *Chlamydia*. Electron-dense nucleoids were readily observed in EB-like particles under histone knockdown conditions. Quantitative TEM analysis further showed that although histone knockdown reduced the proportion of EB-like particles among total chlamydial particles, it did not abolish the formation of EB-like particles with condensed nucleoids. Thus, gross condensation can occur even under conditions of substantial histone depletion. However, progeny EBs generated under histone knockdown conditions were compromised in their ability to efficiently initiate RB growth, indicating that the functionally relevant chromosome features are not captured by morphological condensation alone. Together, these observations support a model in which histone-dependent chromosome architecture, rather than bulk DNA compaction, establishes a state permissive for rapid developmental reactivation.

Our results further demonstrate that HctA and HctB are not functionally equivalent. Deficiency of HctA exerted a dominant effect on subsequent development, whereas loss of HctB alone produced comparatively mild, transient phenotypes by fluorescence imaging without a corresponding reduction in genome copy number. Simultaneous knockdown of both histone genes markedly exacerbated growth impairment, indicating that HctB becomes functionally critical when HctA is absent. These findings suggest a hierarchical relationship in which HctA plays a primary role in establishing chromosome states that support efficient developmental progression, whereas HctB provides additional structural or regulatory support that becomes critical when nucleoid organization is substantially perturbed. These distinctions align with prior heterologous-expression and biochemical studies showing that the two proteins differ in their effects on DNA organization and in their developmental regulation in *Chlamydia* (7–14). Together, the data argue for overlapping but non-identical roles: HctA serves as a principal determinant of chromosome states that prime the genome for reactivation, while HctB supplies complementary support that becomes essential under severe nucleoid perturbation.

The transgenerational consequences of histone deficiency place HctA and HctB within the broader class of bacterial nucleoid-associated proteins (NAPs) that couple chromosome organization to cellular physiology. In many bacteria, NAPs such as HU, IHF, Fis, and H-NS shape large-scale chromosomal architecture and influence global gene-expression programs (21, 22). Experimental work demonstrates that bacterial architectural proteins can directly affect pathogenic behavior (23, 24). Importantly, functional analyses have also linked NAPs to replication dynamics, mutagenesis, and stress-dependent fitness (25). In addition, Lsr2 has been shown to co-condense with AT-rich DNA to form condensates that bridge and compact the chromosome (26). Considered in the context of these prior results, our findings extend the role of bacterial chromosome-architectural proteins beyond static genome organization by linking them to developmental competence across infection cycles in a differentiation-dependent pathogen.

While bacterial epigenetics is frequently discussed in the context of enzyme-mediated marks, for example, DNA methylation and phase variation, our findings point to a protein-centric mode of chromosome memory established by histone homologs. Importantly, earlier biochemical and genetic analyses of the chlamydial HctA and HctB documented DNA binding and transcriptional repression (7–14). Our data implicate histone-mediated chromosome architecture in EBs, consistent with an epigenetic-like mechanism that primes the genome for efficient reactivation following infection (21, 22).

The persistence of nucleoid condensation following simultaneous knockdown of both histone genes indicates that visible genome compaction can occur under conditions of markedly reduced histone expression. However, TEM-based morphology cannot resolve the degree, uniformity, or genomic organization of this compaction relative to wild-type EBs. Thus, preserved electron density should be interpreted as preservation of gross nucleoid condensation, not as evidence that higher-order chromosome architecture is fully normal. These findings point to two non-exclusive explanations: low levels of residual histone protein may suffice for morphological condensation, or additional, yet-to-be-identified DNA-binding factors contribute to EB chromosome packaging, consistent with the broader role of nucleoid-associated proteins in bacterial chromosome organization (21, 22). Future studies using transcriptomic profiling, chromosome-accessibility approaches, nucleoid proteomics, or genome-wide analyses of purified EBs will be needed to determine how HctA and HctB influence local chromosome architecture and transcriptional shutdown during EB maturation. The present findings nevertheless indicate that proper chromosome organization, not gross condensation alone, is required for developmental competence in the next infection cycle.

In summary, our findings redefine the roles of chlamydial histones beyond simple structural mediators of genome condensation. Although HctA and HctB are largely dispensable for EB morphogenesis during the parental developmental cycle, they are critical for establishing chromosome states required for efficient progression through the next infection cycle. These results reveal a previously unrecognized layer of regulation in *Chlamydia* and establish chromosome architecture as a key determinant of developmental fitness across infection cycles.

## MATERIALS AND METHODS

### Plasmids

All inducible CRISPRi vectors targeting *hctA*, *hctB*, or both *hctA* and *hctB* were constructed by assembling PCR fragments amplified from the pL2-idC12-ntg vector (27). To generate pL2-idC12-hctA, an 8,103-bp fragment was amplified using primers hctA-gRNA-F (5′-ATATGGCGCTAAAAGATACGGCAAATTTTTTTGAAGCTTGGGCC-3′) and mKate-R (5′-AATGCGCATTGTTTGAGTT-3′), and a 7,577-bp fragment was amplified using primers mKate-F (5′-CGTATGAAGGAACTCAAACAAT-3′) and hctA-gRNA-R (5′-ATTTGCCGTATCTTTTAGCGCCATATCTACAAGAGTAGAAATTGAAA-3′). The two fragments were fused using the NEBuilder HiFi DNA Assembly Cloning Kit (New England Biolabs) according to the manufacturer’s instructions.

To generate pL2-idC12-hctB, an 8,103-bp fragment was amplified using primers hctB-gRNA-F (5′-ATATCTATCGACAAGGAGAATGAAATTTTTTTGAAGCTTGGGCC-3′) and mKate-R, and a 7,577-bp fragment was amplified using primers mKate-F and hctB-gRNA-R (5′-ATTTCATTCTCCTTGTCGATAGATATCTACAAGAGTAGAAATTGAAA-3′). The two fragments were assembled using the HiFi DNA Assembly Cloning Kit.

To generate pL2-idC12-hctAB, an 8,187-bp fragment was amplified using primers hctAB-array-F (5′-GGAATTGTGAGCGGATAACAATTTCAATTTCTACTCTTGTAGATATGGCGCTAAAAGATACGGCAAAAATTTCTACTCTTGTAGATATCTATCGACAAGGAGAATGAAA-3′) and mKate-R, and a 7,538-bp fragment was amplified using primers mKate-F and Lac-Operator-R (5′-AATTGAAATTGTTATCCGCTCAC-3′). The fragments were assembled using the HiFi DNA Assembly Cloning Kit. Complete plasmid sequences of all constructs were confirmed by Nanopore sequencing at Genewiz (Piscataway, NJ).

### Strains

*C. trachomatis* serovar L2 strain 434/BU was obtained from ATCC and propagated in our laboratory in HeLa cells. EBs were transformed with pL2-idC12-hctA, pL2-idC12-hctB, or pL2-idC12-hctAB to generate L2/hctA-iKD, L2/hctB-iKD, and L2/hctAB-iKD, respectively, as previously described (28). Clonal populations were isolated by limiting dilution (29); EBs were subsequently purified by ultracentrifugation through 35% and 40%/44%/52% MD-76R density gradients (30).

### *C. trachomatis* infection and culture conditions

HeLa229 cells were maintained in Dulbecco’s modified Eagle medium (DMEM) high-glucose (4.5 g/L) supplemented with 10% fetal bovine serum and 20 µg/mL gentamicin at 37°C in 5% CO₂. For gene-expression analyses, growth-rate determinations, and EB-formation assays, cells were seeded into 6- or 12-well plates to form confluent monolayers the following day.

To infect monolayers, EB stocks were diluted in NaHCO₃-free DMEM/F12 (pH 7.4) to achieve a multiplicity of infection of approximately 1 inclusion-forming unit per cell, yielding 80%–90% infection efficiency as assessed by fluorescence microscopy. After the removal of the growth medium, EB-containing inoculum was added to the wells (1.5 mL per well for 6-well plates and 1.0 mL per well for 12-well plates). Plates were centrifuged at 900 × *g* for 30 min at 30°C and then washed three times with DMEM. Completion of the final wash was defined as 0 hpi.

### RNA isolation and RT-qPCR

Total RNA was extracted from infected cells at 24 hpi using TRI Reagent according to the manufacturer’s instructions. Contaminating genomic DNA was removed by two successive DNase I treatments. One-step RT-qPCR with Luna WarmStart Reverse Transcriptase (New England Biolabs) was used to quantify transcript levels of ddCas12, *hctA, hctB*, and *ihtA*. Each sample was analyzed in technical duplicate on a QuantStudio 5 Real-Time PCR System.

Because ATC treatment and histone knockdown did not measurably alter chlamydial genome replication at this time point, 23S rRNA was used for normalization (31). Differential expression was calculated using the ΔΔCt method (25). Primer pairs used for ddCas12, *hctA, hctB, ihtA,* and 23S rRNA were qPCR-dCas12-F (5′-ATGGAACCGTCGTTGAGCTT-3′) and qPCR-dCas12-R (5′-AGGGTCGGCATTTGGAAGTT-3′); qPCR-hctA-F (5′-GGAAATAAAGCCGCAGCAC-3′) and qPCR-hctA-R (5′-ACGATATACCTTCGCGGTCT-3′); qPCR-hctB-F (5′-CATACTGCAGCTTGTGGACG-3′) and qPCR-hctB-R (5′-GCTGTACGAGAACGGTTAGGA-3′); qPCR-ihtA-F (5′-GAGTTGCAAGTTGGTATTCTAACG-3′) and qPCR-ihtA-R (5′-TGTACAAACACTAGAGTCAGAAGC-3′); and qPCR-23S-F (5′-AGATAGACAGCGGGGGCTAA-3′) and qPCR-23S-R (5′-GGTGAGCTGTTACGCACTCT-3′), respectively.

### Sample collection for chlamydial genome copy quantification and IFU assays

At the indicated time points, culture medium was replaced with 500 µL SPG (sucrose–phosphate–glutamic acid solution). Infected cells were detached using cell lifters, collected, and sonicated with a 130-W ultrasonic processor equipped with a 3-mm probe at 35% amplitude for a total of 12 s in alternating 2-s pulses to lyse host cells and release chlamydiae. Lysates were centrifuged at 500 × *g* to remove host-cell debris. An aliquot (200 µL) of the clarified supernatant was used immediately or stored at −20°C for genome copy quantification. The remaining suspension was centrifuged at 20,000 × *g* for 10 min at 4°C to pellet EBs. Pellets were washed twice with 1 mL SPG to remove residual anhydrotetracycline. After the final wash, pellets were resuspended in 200 µL SPG and stored at −80°C for subsequent IFU assays.

### Genomic DNA extraction and relative genome quantification

Chlamydial genome copy number was used as a quantitative proxy for total bacterial burden and RB replication. SPG suspensions of chlamydiae were centrifuged at 20,000 × *g* for 10 min at 4°C. Pellets were resuspended in 100 µL alkaline lysis buffer (0.1 M NaOH, 0.2 mM EDTA), heated at 95°C for 15 min, neutralized with 400 µL 25 mM Tris–HCl (pH 7.2), and stored at −20°C for subsequent analysis, as previously described (27). Relative chromosome abundance was quantified by qPCR using primers targeting *grgA* (5′-GCCGTTTTTACTGCCAGCAT-3′ and 5′-ACTATTGGAGCCACCTTCGG-3′). Each reaction was performed in technical duplicate on a QuantStudio 5 Real-Time PCR System using Power SYBR Green Master Mix. Genome copy differences were calculated using the ΔCt method.

### IFU assays

IFU assays, analogous to colony-forming unit assays for free-living bacteria, were used to quantify infectious EB production. EB stocks were thawed and 10-fold serially diluted in culture medium before inoculation onto 96-well plates containing 3 h-old confluent HeLa229 cell monolayers. Plates were centrifuged at 900 × *g* for 20 min at 25°C and then incubated at 37°C for about 26 h (for plates inoculated with ATC− EBs of all strains and ATC+ EBs of L2/hctB-iKD) or 40 h (for plates inoculated with ATC+ EBs of L2/hctA-iKD and L2/hctAB-iKD). Because all chlamydial transformants used in this study express the far-red fluorescent protein mKate, red fluorescent inclusions were enumerated in live cultures using an Olympus IX-50 fluorescence microscope (20).

### Transmission electron microscopy

Ultrastructural analyses were performed essentially as described previously (20). At 36 hpi, infected HeLa cell monolayers grown in six-well plates were detached using trypsin, collected in phosphate-buffered saline supplemented with 10% fetal bovine serum, and centrifuged for 10 min at 500 × *g*. Cell pellets were resuspended in fixation buffer containing 2.5% glutaraldehyde and 4% paraformaldehyde in 0.1 M cacodylate buffer at room temperature, incubated for 2 h, and stored at 4°C overnight. Samples were rinsed in 0.1 M cacodylate buffer, dehydrated through a graded ethanol series, and embedded in Eponate 812 resin at 68°C overnight. Ninety-nanometer sections were cut using a Leica UC6 ultramicrotome and collected on copper grids. Sections were stained with uranyl acetate followed by lead citrate. Images were acquired as TIFF files on a Philips CM12 transmission electron microscope operated at 80 kV using an AMT XR111 digital camera.

### Fluorescence imaging

EBs harvested from primary cultures grown in the presence or absence of ATC were used to inoculate fresh HeLa monolayers in the absence of inducer. Inclusions were visualized by fluorescence microscopy using the plasmid-encoded mKate reporter present in all strains. Immediately before imaging, culture medium was replaced with phosphate-buffered saline containing calcium and magnesium to reduce background fluorescence. Bright-field and red-fluorescence images were acquired on an Olympus IX51 microscope equipped with an Infinity i8-3 CMOS camera under constant exposure settings. Image overlays were generated using ACINST03 software. The inclusion area and fluorescence intensity were quantified using ImageJ (29).

### Statistical analysis

Statistical comparisons of EB titers, genome copy numbers, and RNA expression between two groups were carried out using two-tailed *t*-tests. Differences in inclusion size and RFP intensity were assessed using the Mann–Whitney test. When appropriate, *P* values were corrected for multiple testing using the Benjamini–Hochberg method to control the false discovery rate. Analyses involving three or more groups were performed by one-way ANOVA with Dunnett’s *post hoc* test. In all figures, one, two, and three asterisks denote *P* < 0.05, *P* < 0.01, and *P* < 0.001, respectively.
